# 

**Published:** 1904-08-15

**Authors:** 


					﻿THE
DENTAL REGISTE
EDITED BY
NELVILLE S. HOFF, D. D.S.,
A MM ADRDD MlfH
15, 1904.	No. 8.
PRICE, ONE DOLLAR A YEAR IN ADVANCE.
PUBLISHED MONTHLY BY
SAMUEL A, CROCKER & CO.,
THE OHIO DENTAL AND SURGICAL DEPOT.
3ö to ííí> W. StlTL St.. 1 íoiiYiiíiti, O.
Entered at the Postoffice at Cincinnati, 0.. as second-class mail matter.
				

